# Neuroimmune Interface in the Comorbidity between Alcohol Use Disorder and Major Depression

**DOI:** 10.3389/fimmu.2016.00655

**Published:** 2016-12-27

**Authors:** Sudan Prasad Neupane

**Affiliations:** ^1^Norwegian National Advisory Unit on Concurrent Substance Abuse and Mental Health Disorders, Innlandet Hospital Trust, Brumunddal, Norway; ^2^Norwegian Centre for Addiction Research (SERAF), University of Oslo, Oslo, Norway

**Keywords:** alcohol use disorder, depression, comorbidity, neuroimmune interface, neuroinflammation, alcohol drinking, cytokines

## Abstract

Bidirectional communication links operate between the brain and the body. Afferent immune-to-brain signals are capable of inducing changes in mood and behavior. Chronic heavy alcohol drinking, typical of alcohol use disorder (AUD), is one such factor that provokes an immune response in the periphery that, by means of circulatory cytokines and other neuroimmune mediators, ultimately causes alterations in the brain function. Alcohol can also directly impact the immune functions of microglia, the resident immune cells of the central nervous system (CNS). Several lines of research have established the contribution of specific inflammatory mediators in the development and progression of depressive illness. Much of the available evidence in this field stems from cross-sectional data on the immune interactions between isolated AUD and major depression (MD). Given their heterogeneity as disease entities with overlapping symptoms and shared neuroimmune correlates, it is no surprise that systemic and CNS inflammation could be a critical determinant of the frequent comorbidity between AUD and MD. This review presents a summary and analysis of the extant literature on neuroimmune interface in the AUD–MD comorbidity.

## Introduction

Alcohol consumption is responsible for 5.9% of global annual deaths and 5.1% of the global disease burden (1). Unipolar major depression (MD) was the second leading cause of years lived with disability worldwide, accounting for 8% of all global years lived with disabilities in 2013 (2). Together, alcohol use disorder (AUD) and MD disorders account for a half of the global disease burden attributable to mental and substance use disorders (3). An unequivocally high comorbidity exists between AUD and MD, with a lifetime comorbidity rate of 20.5% (4). About 30% of individuals with MD report lifetime AUD (5). Conversely, depressive symptoms are common in AUD to the extent that well over a third of AUD patients satisfies diagnostic criteria for MD at some point during their drinking career (6, 7). Compared to isolated disorder, patients with AUD–MD comorbidity carry higher risk of relapse to alcohol dependence, treatment dropout, suicide attempt, and poorer effect of antidepressant medication and have lower global functioning and less life satisfaction (4, 8, 9). Attempts to disentangle causal pathways between depression and AUD have resulted in the wider acceptance of bidirectional causality, with an estimate suggesting one disorder doubles the risk for the other (10). However, the mechanisms of such causality and the interfaces at which they interact remain unclear.

A colloquial understanding of the brain–body interaction is that the brain subjugates the body and pathogenic penetration of the blood–brain barrier is the only route by which bodily immune insults can reach the brain tissue. This view has changed with the demonstration of immune signals in the form of inflammatory cytokines that access the brain *via* afferent vagal fibers (11), by directly crossing leaky regions in the blood–brain barrier (e.g., area postrema), through cytokine-specific active transport molecules and through secondary messenger molecules within the CNS endothelia (12). Microglia and astrocytes can in turn accentuate CNS cytokine load. These cytokines and the relayed signals in the brain interact with various neurotransmitter systems as well as the hypothalamic–pituitary–adrenal (HPA) axis, the primary hormonal response system to stress (13). Furthermore, co-stimulatory signals that allow mast cells to interact with the immune cells and influence the integrity of the blood–brain barrier are important mediators of the cross talk between the peripheral and the central neuroimmune signaling (14). Thus, immune inflammatory signals in the brain are key to the translation of psychological and biological stressors into behavioral outcomes.

Several lines of research show both AUD and MD are, as isolated disorders, associated with various changes in immune function. There is, however, a paucity of knowledge on the role of neuroimmune function in the development and progression of comorbid AUD and MD. As an example, a binge pattern of drinking is particularly depressogenic (10), but the exact underlying neurobiological mechanism for this “alcoholic depression” awaits elucidation. The available evidence indicates that allostatic changes in the neuroimmune functioning could have significant impact on the development, progression, and outcome of AUD–MD comorbidity, and promising neuroimmune targets are being identified to address these issues. Several caveats remain before these developments in psychoneuroimmunology of comorbid psychiatric disorders could be capitalized.

## AUD and Immunity

Alcohol is a potent modulator of the immune system and alters the expression of inflammatory mediators in the periphery as well as in the CNS. A well-described mechanistic explanation for this is that heavy alcohol consumption activates toll-like receptor (TLR) systems, including the TLR2 and TLR4 (15), through the danger-associated molecular pattern signaling, which renders the gut wall “leaky” then enabling the translocation of microbial products such as lipopolysaccharides (LPS) into circulation. This effect has been confirmed both in binge drinking (16) and chronic heavy drinking among humans (17, 18) and more widely in animal models (19, 20). The leaked LPS potentiates alcohol-induced liver inflammation and stimulates immune cells such as monocytes, macrophages, T lymphocytes, and dendritic cells to cause the release of pro-inflammatory cytokines, including interleukin (IL)-1β, IL-6, and tumor necrosis factor-alpha (TNF-α) (21). Peripherally produced cytokines and chemokines [e.g., monocyte chemoattractant protein-1 (MCP-1)] and/or their signals eventually relay to multiple brain regions, where they further activate brain microglia and astrocytes to produce CNS cytokines. The cytokine production in the brain is again dependent on TLR4 signaling and is propagated along the mitogen-activated protein kinase and NF-κB pathways. It appears that alcohol-induced cytokine upregulation follows the pattern of LPS but with less intensity. Within an hour of an intoxicating dose (5 g/kg) of ethanol, IL-10 levels were already significantly increased in rat hippocampus (22). Qin and colleagues demonstrated that comparable doses of ethanol in binge and chronic alcohol drinking paradigm in mice could induce IL-1β, TNF-α, and MCP-1 production in the liver, plasma, and brain tissues (23). In the liver and other peripheral organs, cytokine upregulation upon LPS or alcohol resolves within days to weeks. Remarkably though, brain immune activation induced by ethanol, or by LPS upon sensitization with ethanol, persisted for many months (23, 24). Using postmortem brain samples, the same group discovered that MCP-1 concentrations were increased in the ventral tegmental area, substantia nigra, hippocampus, and amygdala of alcoholic brains compared to the MCP-1 concentrations in those brain areas of moderate drinking controls (25). Since these areas are relevant to reward, emotion, and behavioral functions, MCP-1 is potentially involved in the neurodegenerative pathologies of alcohol. It is at this juncture that alcohol-induced neuroinflammation becomes clinically relevant because persistent neuroinflammation clearly precipitates cognitive and behavioral responses (26). It has recently been proposed that neuroimmune signaling is an important contributor to the development and maintenance of alcohol dependence (27). Thus, the enduring nature of the neuroimmune induction in the brain resonates with the chronicity of alcohol addiction and might represent a mechanism contributing to the development of closely comorbid conditions of alcohol dependence, such as depression (23, 24).

**Figure 1:**
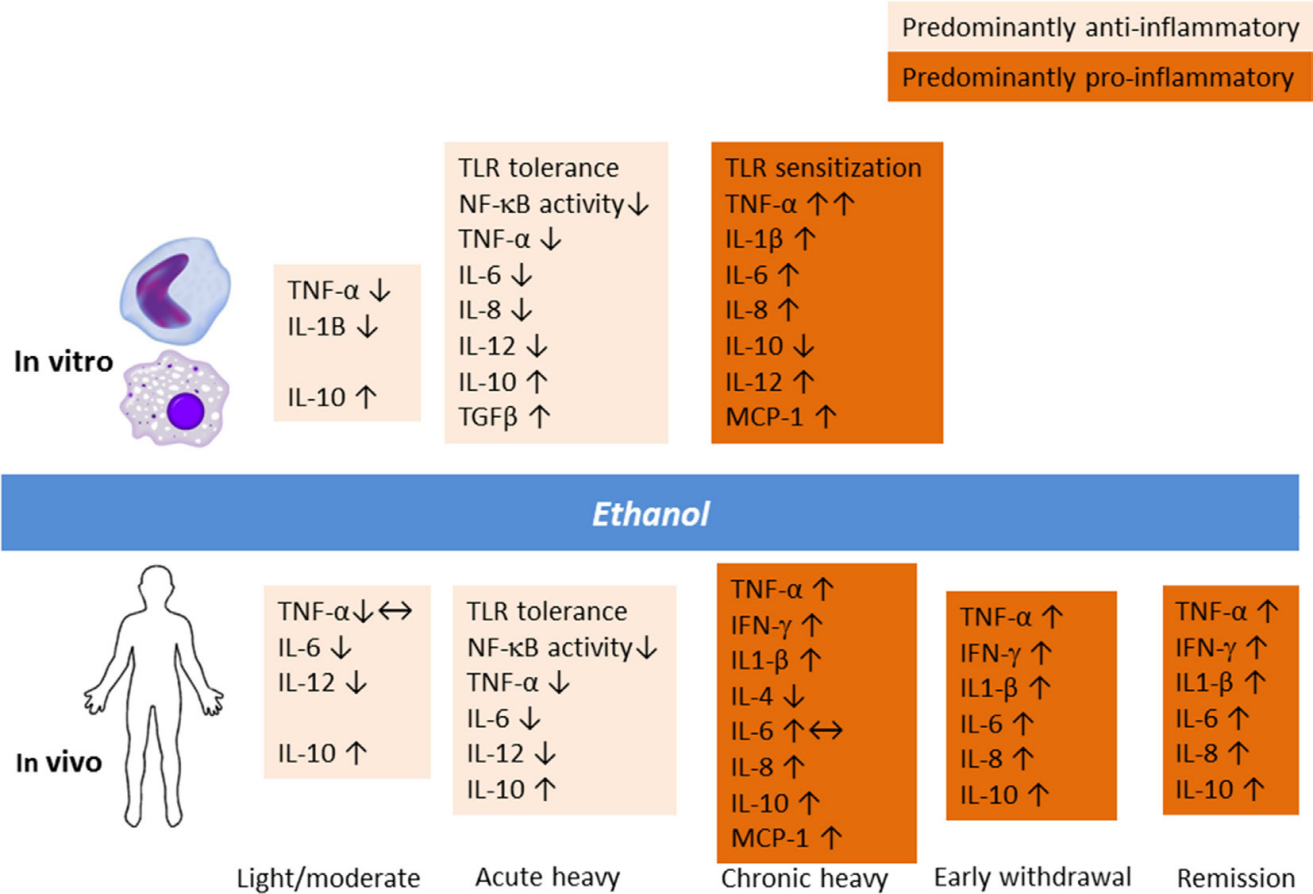
**Alcohol modulation of the innate immune response *in vitro* and *in vivo* involves a complex dynamic depending upon the dose and duration of exposure and chronicity of alcohol use and alcohol use disorder**.

Alcohol modulation of the immune system involves a complex dynamic dependent on the dose and duration of exposure and chronicity of AUD (Figure 1). Acute heavy alcohol consumption (e.g., ≥3 g/kg), even in a single dose, inhibits inflammatory cell activation (28–30). Upon LPS challenge, alcohol-primed mice suppressed lung TNF-α activity, TNF-Rp55 mRNA expression, and soluble TNF-Rp55 levels (31). Ethanol suppressed LPS-induced expression of IL-1, IL-6, and their receptors while significantly upregulating IL-10 levels. In fact, acute ethanol blunted LPS-induced TNF-α secretion by 40%. This immune suppressing effect of alcohol drinking has long been appreciated. The exact molecular mechanism for the opposing immune effects of acute and chronic alcohol remains unclear. However, alcohol-induced tolerance and sensitization of TLRs depending on the length of exposure to alcohol may play a role. Through a series of experiments on human monocytes stimulated with LPS and on animal binge drinking models, Szabo and colleagues demonstrated that acute alcohol induces TLR4/LPS tolerance through activation of a nuclear protein Bcl-3, which interacts with the p50 subunit of the nuclear factor kappa-light-chain-enhancer of activated B cells (NF-κB) (32). The Bcl-3–NF-κB p50 interaction results in the suppression of transcription of NF-κB-regulated genes, including that of pro-inflammatory cytokines (33). Furthermore, chronic alcohol switched the anti-inflammatory response to a pro-inflammatory response by human monocytic sensitization to LPS through decreased expression of interleukin-1 receptor-associated kinase-M, a negative regulator of TLR signaling, and subsequent activation of NF-κB, an effect opposite to acute alcohol (34).

Animal studies have demonstrated that an anti-inflammatory effect of acute binge drinking ensues already in the first hour and lasts beyond ethanol elimination from the body (22, 31). The same finding was replicated in humans 13 h after intake of 1.5 g/kg of alcohol (35). The experiment showed that stimulated peripheral blood mononuclear cell production of IL-10 and IL-12 as well as IFN-γ was increased upon early withdrawal. Thus, the continuum of heavy drinking, withdrawal, craving, and relapse to alcohol use potentially involves immune inflammatory signaling, an area that deserves further investigations. Recently, a study of rat models of acute alcohol intoxication suggested that the expression of inflammatory cytokines is elevated during the withdrawal phase, but changes in the central nervous system appeared to be site dependent (36). In particular, IL-6 levels were increased in multiple brain regions following alcohol exposure and lasted for up to 18 h. Thus, acute heavy drinking favors apoptotic and anti-inflammatory changes (37, 38), whereas chronic heavy drinking is known to induce monocytic TNF-α production as well as T- and B-cell activation (38, 39).

Clearly, the neuroimmune and endocrine modulatory function of alcohol varies depending on whether the individual is a social drinker or has a severe AUD (Figure 1). Gonzalez-Quintela et al. (40) reported that, among Spanish adult men and women, light-to-moderate alcohol drinking was not associated with altered levels of TNF-α. In clinical AUD populations, however, levels of cytokines are typically increased compared to non-drinking individuals (26, 41, 42). A recent study from Taiwan showed that the levels of inflammatory cytokines were elevated during the early withdrawal phase (up to 4 days of abstinence), which was considerably ameliorated upon 4 weeks of abstinence (43). Regarding alcohol effects on HPA axis, the findings have been controversial, but varying results probably indicate different vulnerability factors and the extent of familiarization with alcohol (44). Corticosterone levels surges following acute alcohol intake by social drinkers in a dose-dependent manner, but this response is dampened in chronic AUD (45). In clinical AUD populations even without liver disease, levels of cytokines are typically increased compared to non-drinking individuals (26, 41, 42). As I will elaborate later, pro-inflammatory responses seen in chronic alcohol misuse are akin to those seen in MD.

Recent studies have shown long-term negative health outcomes in animals exposed to ethanol prenatally. In adult rats prenatally exposed to ethanol, the corticosterone reservoir was depleted and the cytokine production upon immune challenge was exaggerated (46). The ensuing low-grade inflammation correlated with memory deficits, which implicated a microglial role in fetal alcohol spectrum disorders (47). Also, substantial neuroinflammation caused by traumatic brain injury induced escalation of drinking in ethanol-habituated animals (48). Thus, CNS inflammation in both low and high grades changes alcohol drinking behavior. This phenomenon is clinically relevant.

Human experimental studies on the immune effects of ethanol consumption in healthy individuals are rare for obvious ethical reasons. A few endeavors have confirmed an early recruitment of immune cells following ethanol intoxication. Afshar et al. (49) gave a binge dose of alcohol to healthy men (0.9 g ethanol/kg body weight) and women (0.8 g ethanol/kg body weight) and found a surge in the number of circulating monocytes, leukocytes, and natural killer cells—within 20 min of alcohol intake, which was followed by recovery toward baseline within 5 h. Thus, both innate and adaptive arms of the immune system are affected by alcohol. LPS induction of whole blood in the same sample showed fluctuations in inflammatory cytokines, and at 5 h, an anti-inflammatory state set in with elevated IL-10 and reduced IL-1β levels. No sex differences in immune response were reported, although animal studies indicate that females are more vulnerable to the neuroinflammatory effects of alcohol (50). For example, chronically ethanol-treated female mice expressed relatively greater levels of inflammatory mediators (iNOS and COX-2), cytokines (IL-1β, TNF-α), gliosis processes, caspase-3 activation, and neuronal loss in the cerebral cortex compared to their male counterparts (50). This finding was confirmed in postmortem brain specimens of AUD individuals who had higher MCP-1 levels and increased microglial activation markers compared to controls (25). Human pharmacogenetic studies on alcohol dependence have coincidentally discovered several immune-gene polymorphisms as underlying excessive drinking (51).

## Neuroimmune Alterations in Depression

The identification of immune disturbance in depressive illness (52) led to the “macrophage hypothesis of depression,” the proposition that inflammatory products of macrophage were responsible for depression (53). Since then, a consistent body of literature has confirmed that inflammatory processes are involved in the development and progression of depressive illness. Numerous studies have consistently documented positive associations of MD with C-reactive protein (CRP) and IL-6 (54). Meta-analyses have also supported depression’s associations with IL-1β (55) and TNF-α (56, 57) as well as sIL-2 receptor (57). These associations held true for patient populations from the community as well as from clinical inpatient/outpatient settings. Patients with depression are found to have renormalized cytokine levels following treatment (58). Furthermore, several reports indicate longitudinal associations between CRP and subsequent development of depression (59), although an association was found in the opposite direction in a younger sample (60). Compelling evidence exists to suggest elevated levels of IL-6 as both a cause and a consequence of depression (61). In a 12-year prospective study of British civil servants, increased IL-6 levels at baseline predicted cognitive symptoms of depression at follow-up (62). These effects were reported to be consistent even after accounting for possible confounders such as, socio-demographics, behavioral and biological risk factors, health conditions, medication use, and baseline negative emotions. Recently, a population-based study from England (*N* = 5,909) showed positive associations between CRP and symptoms of fatigue, disturbed sleep, low energy, and low mood in a dose–response manner, a relationship that was absent in antidepressant medication users (63).

These novel findings quickly triggered drug trials using anti-inflammatory agents in depressive illness in humans. Notably, a proof-of-concept study examined infliximab, a TNF-α blocker in patients with treatment-resistant depression. Twelve weeks after the initiation of therapy, infliximab reduced depressive symptoms by at least a half among patients with baseline hs-CRP > 5 mg/L, but not among those with lower baseline hs-CRP levels (64). Yet, another trial showed that adjunctive celecoxib, a selective COX2 inhibitor, was more effective in reducing depressive symptoms than sertraline alone in MD (65). Again, a reduction of serum IL-6 levels correlated very well with a reduction in depression score. However, the observation period was only 6 weeks. Several other drug trials using non-steroidal anti-inflammatory drugs have been conducted (66), mostly without rigorous patient selection. Significant methodological heterogeneity and publication bias make the reported positive efficacy less tenable.

One mechanism by which activated inflammatory cytokines (mainly IFN-γ and TNF-α) can aggravate depressive symptoms is through their induction of indolamine 2,3-dioxygenase (IDO), an enzyme that metabolizes tryptophan along the neurotoxic kynurenine pathway (67, 68). IDO induction causes relative reduction in the availability of tryptophan, which is the amino acid precursor for serotonin synthesis. Tryptophan depletion and the neurotoxic metabolites produced downstream the kynurenine pathway may both trigger depression. In particular, peripheral macrophages and brain microglia preferentially metabolize kynurenine into anthranilic acid and quinolinic acid, both of which are NMDA receptor agonists and have potentially neurotoxic effects (69). Approximately, half of the cancer patients treated with IFN-α immunotherapy develops depression, and it was found that the severity of IFN-α-induced depression was related to the tryptophan degradation index (kynurenine to tryptophan ratio) along the kynurenine pathway (70). Studies also show a higher tryptophan degradation index ratio in individuals with MD compared to healthy controls (71, 72).

The failure of monoamine hypothesis to explain the delayed symptom relief in depression, despite early changes in brain monoamine neurotransmitter concentration following treatment, led to the emergence of the neurotrophic hypothesis of depression. It posits that chronic stress leads to reduced neurotrophic support to the brain limbic structures responsible for regulating mood and increases vulnerability to depression (73). Indeed, numerous studies have shown reduced serum levels of brain-derived neurotrophic factor (BDNF) in patients with MD compared to healthy controls (74–76), and evidence also exists to support renormalization of BDNF levels upon successful anti-depression interventions (74, 77). This process takes weeks to months. CNS and peripheral BDNF concentrations are altered in several mood and behavioral aberrations (74, 75, 78). Overexpressed pro-inflammatory cytokines in the brain and associated chronic neuroinflammation can lead to neurodegeneration and reduced neurogenesis, as indicated by decreased BDNF in multiple brain areas following LPS challenge (79). However, the cytokine network is rather complex, including pleiotropic effects that are sometimes paradoxical. For example, both neurotrophic and neurodestructive properties of IL-6 have been reported (80). Accordingly, circulating BDNF levels in depressed individuals were positively correlated with IL-6, but not with TNF-α (81). In recovering alcoholics, however, serum BDNF levels were positively correlated with IL-6 and TNF-α (82). Thus, the interaction between inflammatory cytokines and BDNF remains an active area of research.

While the search for neuroimmune targets in depression continues, alternative medicine has also contributed to the field. Salidroside, a traditional Tibetan herbal product, known for its antioxidative and immunotonic effects, was administered to mice that were later exposed to LPS (83). The study revealed that salidroside could effectively ameliorate LPS-induced depression-like behavior while also attenuating the inflammatory cytokine and NF-κB. Further investigations using polyphenolic compounds such as curcumin and resveratrol are underway to test the possible role of these agents in HPA axis modulation, hippocampal neurogenesis, and central monoamine homeostasis. Additionally, several other compounds related to immune regulation are of value: statins, polyunsaturated fatty acids, ketamine, TLR-inhibitors, glycogen synthase kinase-3 inhibitors, oleanolic acid analogs, and minocycline (84).

The most pressing caveat is that inflammation is neither necessary nor sufficient to cause depression, which means that activated inflammatory response would accompany only a subgroup of individuals with MD. Circulating and CNS levels of the inflammatory cytokines induced by alcohol are also modest, typically exceeding the levels in the healthy controls by a factor of 2–5 (24, 43). Unlike in purely inflammatory conditions, inflammatory markers in these low-inflammatory states rise only marginally, thus making interpersonal variations difficult to interpret (85). Nonetheless, a finding of sustained immune activation can connect depression as well as AUD with the often coexisting conditions of low-grade inflammation such as cardiovascular diseases, diabetes, fibromyalgia, multiple sclerosis, and cancer (86). A bulk of psychoneuoroimmunological literature stems from correlational evidence, which is clearly inadequate to explain the depression pathophysiology and to subsequently proffer clinical interventions. Thus, it is high time that the theoretically embraced entity of “inflammatory cytokine-associated depression” (87) be phenotype based on relevant biological and clinical characteristics. Omics-based approaches highlighting systems biomedicine could be beneficial (88). Only such progress would lead to an enhanced understanding of comorbid conditions of MD.

## Neuroimmune Dysregulation in AUD–Depression Comorbidity

**Figure 2:**
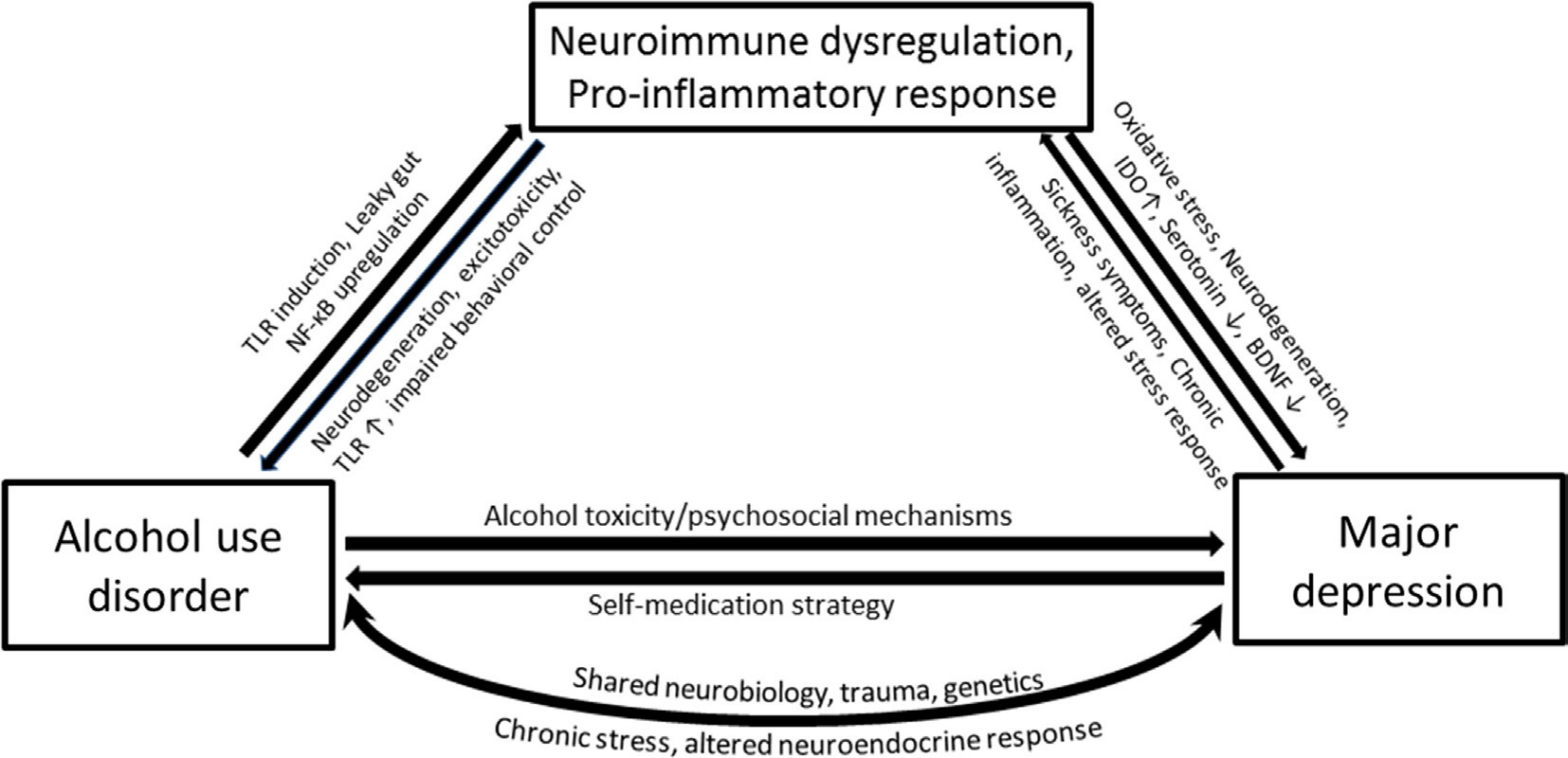
**Simplified pathways illustrating the potential mechanisms that underlie associations between alcohol use disorder (AUD) and major depression (MD), with a compelling neuroimmune contribution**. Heavy alcohol drinking may render gut wall permeable to bacterial proteins such as lipopolysaccharide (LPS) through the activation of toll-like receptors. Ethanol and LPS upregulate the transcription factor NF-κB and cause immune cells in the periphery as well as glial cells to produce pro-inflammatory cytokines. The pro-inflammatory cytokines within the brain activate the indolamine 2,3-dioxygenase enzyme, which metabolizes tryptophan away from serotonin production toward a potentially neurotoxic kynurenine pathway. MD accompanies altered monoamines, oxidative and nitrosative damage, and neurodegeneration. Depression is associated with chronic inflammatory conditions such as cancer and cardiovascular diseases, which together with sickness symptoms can feed the neuroimmune dysregulation causing further neurodegeneration, negative affect, and anxiety-like behavior and loss of behavioral control—all features characterized in AUD.

It should be noted that immune perturbations presented in the previous sections that focused on inflammatory cytokines are only parts of several interacting biological systems that are ascribed to AUD and MD. The proposed interrelated inflammatory and neurodegenerative mechanisms responsible for the neurobiological changes in depression involve activated central and peripheral pro-inflammatory cytokine response, lowered levels of zinc and ω3 polyunsaturated fatty acid overload, oxidative and nitrosative stress, tryptophan degradation along the kynurenine pathway, reduced neurogenesis, and increased neurodegeneration (89). A complex interaction between these processes produces neurobiology of depression and contributes to related brain disorders. For example, inflammatory cytokines in the brain are toxic to dopaminergic neurons and may precipitate Parkinson’s disease (90). Two main factors contributing to the development of alcohol addiction are reinforcement (positive and negative) and neuroadaptation, both of which seem closely related to alterations in these processes, as has been elaborated in previous sections. Given the high rates of comorbidity and overlapping pathophysiological changes in various aspects of the neuroimmune system that accompany each disorder, it will be no surprise if neuroimmune changes in AUD–MD comorbidity are somehow coordinated (Figure 2).

Hypothalamic–pituitary–adrenal-axis hyperactivity and glucocorticoid receptor impairment are reliable findings in depression (91), and altered HPA axis regulation is a hallmark of hormonal dysbalance in AUD (92). The nature of HPA axis abnormality upon ethanol depends on various stages of the disease and ethanol dose. In chronic AUD cases, basal ACTH levels are elevated and stress- and cue-induced corticotropin and cortisol responses are suppressed (93). Alcohol withdrawal syndrome is characterized by symptoms of autonomic hyperactivity such as tremor, sweating, anxiety, agitation, nausea, and malaise. Symptoms also include disturbed sleep and depressed mood. Interestingly, blocking the hypothalamic corticotropin-releasing factor (CRF) ameliorates the dysphoric symptoms of alcohol withdrawal (94) and the increased stress responsiveness and associated anxiety-like behavior during protracted abstinence (95). CRF blockade in depression-like behavior in a mouse model was shown to reduce those symptoms through modulation of neuronal plasticity (96). Taken together, involvement of brain stress systems in neuroadaptive changes accompanying addiction and emotional circuitry provides a common interface for AUD- and MD-related neuronal changes.

Inflammatory cytokines are potent inducers of CRF and, therefore, negative affect during withdrawal and negative reinforcement during long-term abstinence could potentially arise from immune-mediated CRF activation. Glucocorticoids thus produced cause tryptophan degradation by activating hepatic degradation of tryptophan 2,3-dioxygenase (TDO), which, along with cytokine-induced IDO in the brain, once again produces metabolites biased toward the neurotoxic edge (97, 98). TDO enzyme is activated upon acute alcohol consumption, subsequently inhibited with chronic alcohol drinking, and again surges during ethanol withdrawal (99–101). The altered tryptophan metabolism reportedly lasts for several months into abstention, as shown in a comparative study of 4 and 11 weeks of abstinence, wherein longer abstinence was related to increased kynurenine levels (102, 103). This could well be explained in terms of hyperactive stress response in concert with negative reinforcement, craving, and relapse. We reported increased tryptophan turnover with increased duration of abstinence (104). However, another study (105) showed that AUD individuals who abstained from alcohol for longer than two weeks, regardless of background variables, had much higher tryptophan levels compared to healthy controls. Literature also indicates a contradictory higher tryptophan and lower tryptophan degradation in depression, alongside activated pro-inflammatory pathway (104, 106, 107), but these findings are based on peripherally measured mediators and may not reflect brain levels. An overview of the few studies that have investigated neuroimmune mediators in the context of AUD–MD comorbidity are shown in Table 1. Further studies are needed to clarify these observations.

**Table 1:** **Overview of studies investigating neuroimmune pathways in comorbid alcohol use disorder (AUD) and depression**.

Reference, country	Subjects	Studied pathway/parameter	Main findings
Han et al. (110), South Korea	45 male inpatients with alcohol dependence	Growth factors	Depression score in AUD patients correlated positively with insulin-like growth factor, but not with nerve growth factor or BDNF
Plemenitaš et al. (122), Slovenia	101 alcohol abusing and 100 previously alcohol-dependent male inpatients abstinent for ≥2 years	Tryptophan metabolism; genetic association study	Genetic variability in tryptophan hydroxylase 2 (TPH2) gene associated with anxiety and, to some extent, with depression. TPH2 rs1843809 was associated with depressive and aggressive traits and TPH2 rs4290270 with depressive and anxiety traits
Neupane et al. (104), Nepal	153 male and 16 female AUD inpatients	Tryptophan metabolism	Concurrent depressive state related to counterintuitive higher tryptophan level and lower tryptophan degradation index. Tryptophan metabolism related to abstinence duration and AUD severity
Neupane et al. (82), Nepal	152 male AUD inpatients	BDNF	Concurrent depressive state in AUD related to lower BDNF serum levels. Among patients in controlled abstinence, history of binge drinking, and severe AUD associated with higher BDNF serum levels. Tumor necrosis factor-alpha (TNF-α) correlated with BDNF levels
Neupane et al. (107), Nepal	156 male and 20 female AUD inpatients	Cytokines	Higher serum levels of inflammatory cytokines [interleukin (IL)-6, TNF-α, IFN-γ], but not IL-10 among comorbid major depression (MD) group. Cytokine levels less increased in depression comorbid with greater severity of AUD than less severe AUD
Nedic et al. (111), Croatia	549 male and 126 female patients with alcohol dependence	BDNF; genetic association study	BDNF Val66Met polymorphism not related to depression in alcohol dependence
Su et al. (112), China	548 male Han Chinese with alcohol dependence	BDNF; genetic association study	The A allele of BDNF rs6265 was significantly overrepresented in alcohol-dependent patients with depression compared to patients with isolated alcohol dependence
Umene-Nakano et al. (113), Japan	13 male and 6 female inpatients with MD and alcohol dependence	BDNF	No significant difference was found in the serum BDNF levels of depressive patients with and without alcohol dependence. BDNF levels increased among responders to antidepressant medication (8 weeks), but not among non-responders

*BDNF, brain-derived neurotrophic factor*.

Neurotrophic changes in brain regions involved in depression and AUD are relevant considerations. As discussed in the previous section, depression is consistently associated with depleted BDNF. Indeed, neuroinflammation has an inhibitory effect on adult cortical and hippocampal neurogenesis, as evidenced by reduced BDNF expression concurrent to LPS-induced upregulation of inflammatory cytokines in rats (79). Similarly, chronic ethanol exposure in humans was accompanied by reduced BDNF expression in the hippocampus (108) as well as lower plasma BDNF protein levels (109). Reduced expression of BDNF in the hippocampus and cortical regions is a clear conjuncture for AUD and depression because these are critical target brain regions in both disorders. During the last decade, we (82) and others (110–113) have investigated BDNF in AUD patients. The findings of these studies indicate that neuronal repair initiates soon after the abstention commences, and BDNF levels continue to rise over several months (109, 113–117). Rat models of alcoholism showed that augmenting BDNF actions by the use of the selective BDNF tyrosine kinase B receptor agonist (7,8-dihydroflavone) removed withdrawal-induced depression-like behavior (118). Depressive symptoms are observed during various stages of AUD. In many cases of AUD, associated depressive symptoms do not disappear even after sustained abstinence. Against this backdrop, bidirectional causality between AUD and MD has been demonstrated with a more robust association seen from AUD leading toward MD (10). These evidences suggest the existence of what might be considered *alcoholic depression* and that a biological explanation for depression in AUD could be approached from the immune inflammatory and stress pathways. It remains to be shown how the neuroadaptive changes in recovering AUD individuals relate to depressive symptoms, and whether targeting key neuroimmune factors such as BDNF is a viable intervention option in AUD–MD comorbidity.

Immune signaling induces a range of physiological responses that are common to affective and behavioral disorders. Infection accompanies a TLR4-mediated pro-inflammatory response, indicated by raised IL-1β, IL-6, and TNF-α levels, which leads to “sickness behavior” (119, 120). Sickness behavior is also observed upon psychological stress and exogenous cytokine administration such as during cancer treatment with IFN-α and includes physiological responses (e.g., fever and disturbed sleep) as well as behavioral symptoms (e.g., anorexia, reduced mobility, disappearance of body care activities and reduced social interaction) (119). Many of these features overlap with those of depression. Compelling evidence also suggests activated TLR4 signaling to accentuate alcohol drinking but also negative affect and anxiety-like behavior, especially during the withdrawal phase (121). Sickness symptoms wane away over several days; however, cytokine induction of these behavioral changes may persist as MD. Thus, a better understanding of the loop between immunity, the brain, and behavioral outcomes holds promises to newer approaches to intervene AUD–MD pathologies.

## Conclusion and Future Directions

The clinical realm of frequent comorbidity between AUD and MD requires an integrated psychobiological understanding that underlies these conditions. As presented in this review, mounting evidence supports neuroimmunological alterations, in particular activated immune responses (26), as a critical piece of the physiological link between AUD and MD. Neuroimmune gene induction in limbic brain regions increases negative affect, drug-seeking behavior, and loss of behavioral control (51). Diminished affect is a hallmark of depression, and anxiety-like behavior is pronounced in the withdrawal phase of alcohol addiction. Thus, mood symptoms as well as emotional and behavioral lability in AUD and MD appear to stem from neuroimmune mechanisms (21, 51, 87). The relative contribution of one phenomenon in the context of the other remains unclear. The current evidence is clearly inadequate to unravel the full scope of possible neuroimmune etiopathology of isolated AUD and MD, and an endeavor to attack their comorbidity may sound premature at this stage. However, this complexity should not be a hindrance to investigate these two disorders in their totality, because the relative neuroimmune contribution to each disorder may become clearer when one of the comorbid conditions vanishes. Prospective studies investigating longitudinal associations between changes in neuroimmune function and changes in depressive symptoms, drinking behavior, and treatment outcome are necessary. Furthermore, researchers in the field should be aware of the ethical obligation not to categorically exclude patients who have additional burden.

An extension of the research focus from isolated to comorbid disorders and from preclinical to clinical settings is conducive to appraising the significant overlaps in manifestation of AUD and MD, as well as common biological perturbations in their development and maintenance. It is important to note that the neuroimmune approach alone would not be sufficient to elucidate the underlying complex etiopathology of AUD and MD, which strongly involves other genetic, epigenetic, and environmental factors. However, the neuroimmune approach would constitute an essential component of the systems biomedicine and be applicable to a significant proportion of patient populations. Equally important is the identification of intermediary processes that may determine the ultimate neuroimmune allostasis. Taken together, an exploration of a neuroimmune model for AUD–MD comorbidity provides a foundation for the development of more effective immune-based pharmacotherapy against these burdensome disorders.

## Author Contributions

The author confirms being the sole contributor of this work and approved it for publication.

## Conflict of Interest Statement

The author declares that the research was conducted in the absence of any commercial or financial relationships that could be construed as a potential conflict of interest.
